# Cladodionen Is a Potential Quorum Sensing Inhibitor Against *Pseudomonas aeruginosa*

**DOI:** 10.3390/md18040205

**Published:** 2020-04-10

**Authors:** Mengjia Wang, Lu Zhao, Hao Wu, Chaoyue Zhao, Qianhong Gong, Wengong Yu

**Affiliations:** 1 School of Medicine and Pharmacy, Ocean University of China, 5 Yushan Road, Qingdao 266003, China; wangmengjia6877@stu.ouc.edu.cn (M.W.); zhaolu@stu.ouc.edu.cn (L.Z.); wuhao1330@stu.ouc.edu.cn (H.W.); zhaochaoyue2712@stu.ouc.edu.cn (C.Z.); 2 Laboratory for Marine Drugs and Bioproducts, Qingdao National Laboratory for Marine Science and Technology, 1 Wenhai Road, Qingdao 266237, China; 3 Provincial Key Laboratory of Glycoscience and Glycotechnology, Ocean University of China, 5 Yushan Road, Qingdao 266003, China

**Keywords:** marine fungi, *Cladosporium* sp. Z148, cladodionen, quorum sensing inhibitor, *Pseudomonas aeruginosa*

## Abstract

*Pseudomonas aeruginosa* is an opportunistic pathogen using virulence factors and biofilm regulated by quorum sensing (QS) systems to infect patients and protect itself from environmental stress and antibiotics. Interfering with QS systems is a novel approach to combat *P. aeruginosa* infections without killing the bacteria, meaning that it is much harder for bacteria to develop drug resistance. A marine fungus *Cladosporium* sp. Z148 with anti-QS activity was obtained from Jiaozhou Bay, China. Cladodionen, a novel QS inhibitor, was isolated from the extracts of this fungus. Cladodionen had a better inhibitory effect than pyocyanin on the production of elastase and rhamnolipid. It also inhibited biofilm formation and motilities. The mRNA expressions of QS-related genes, including receptor proteins (*lasR, rhlR and pqsR*), autoinducer synthases (*lasI, rhlI and pqsA*) and virulence factors (*lasB and rhlA*) were down-regulated by cladodionen. Molecular docking analysis showed that cladodionen had better binding affinity to LasR and PqsR than natural ligands. Moreover, the binding affinity of cladodionen to LasR was higher than to PqsR. Cladodionen exhibits potential as a QS inhibitor against *P. aeruginosa*, and its structure–activity relationships should be further studied to illustrate the mode of action, optimize its structure and improve anti-QS activity.

## 1. Introduction

*Pseudomonas aeruginosa* is an opportunistic pathogen causing a wide range of acute and chronic infections in cystic fibrosis patients, immunocompromised individuals, burn victims and patients who are mechanically ventilated [1]. Antibiotics cause enormous selective pressure by killing *P. aeruginosa* or inhibiting its growth, leading to the development of drug resistance in this bacterium. Virulence factors and multiple mechanisms, including the formation of a biofilm, are also used by *P. aeruginosa* to infect hosts and protect itself from environmental stress and antibiotics [2]. The production of virulence factors and the formation of biofilms are under the control of quorum sensing (QS), a process of cell-to-cell communication to regulate group behaviors [3]. Compared with traditional antibiotics, interfering with QS systems is a novel means to effectively reduce virulence and combat *P. aeruginosa* infections with less selective pressure, meaning that it is much harder for the bacteria to develop drug resistance. 

There is a hierarchy QS network which plays a key role in the regulation of the expression of virulence genes and the formation of biofilms in *P. aeruginosa* [3]. At the top of the hierarchy QS network, the *las* system governs the expression of the other QS systems, including *rhl*, *PQS* and *IQS*, of *P. aeruginosa* [3]. As the second positive feedback loop, the *rhl* system is regulated not only by the *las* system, but also by the *PQS* system [4]. The *PQS* system is the third QS system mediated by quinolone signal molecules. The fourth QS system, *IQS*, could partially take over the functions of the *las* system under phosphate depletion stress conditions [5]. Many virulence factors that play an important role in the infection of *P. aeruginosa* are regulated by QS systems. Elastase and rhamnolipid are mainly regulated by the *las* and *rhl* systems, mediated by *N*-acyl homoserine lactones (AHLs) [6]. Pyocyanin is mainly regulated by the *PQS* system [7]. Biofilms are a common cause of persistent infections and are regulated by QS systems [8,9,10]. Therefore, QS inhibitors (QSI) present a promising alternative to manage *P. aeruginosa* infections by reducing virulence.

The LuxR-type receptor proteins LasR and RhlR are activated by AHLs, while the LysR-type transcriptional regulator PqsR is activated by quinolone signal molecules. To date, the crystal structure of RhlR has not been determined. Currently available crystal structures of LasR contain a ligand binding domain (LBD), which is highly soluble in the presence of *N*-(3-oxododecanoyl)-l-homoserine lactone (3-oxo-C12-HSL) [11]. The symmetrical dimer structure is essential for the LasR to exert its transcriptional regulatory activity, with each monomer exhibiting an α-β-α fold. Amino acid residues Tyr56, Ser129, Trp60 and Asp73 make H-bonds to 3-oxo-C12-HSL which are housed in a large hydrophobic pocket formed by 33 residues [11,12]. PqsR (MvfR) is a key component of alkyl-quinolone-dependent QS in *P. aeruginosa* [13]. The LBD of PqsR consists of two subdomains connected by an antiparallel β-sheet hinge region, with a large ligand binding pocket in which a native agonist 2-nonyl-4-quinolone (NHQ) is stabilized entirely by hydrophobic interactions [13,14]. Residues Leu189, Leu207, Leu208, Ala168, Ile149, Ile236, Tyr258, Ile186 and Val170 take part in the above hydrophobic interactions between PqsR and NHQ.

The marine environment accounts for about 95% of the biosphere in the world, and is the most abundant and diverse source of new drugs [15]. Marine fungi represent quite a diverse group and have huge potential for new natural products [16]. Considering the widespread interactions between bacteria and fungi, it is possible to find inhibitors against bacterial QS systems from secondary metabolites of marine fungi. Herein, we obtained marine fungus *Cladosporium* sp. Z148 with anti-QS activity from Jiaozhou Bay, China. The bioactive compound isolated from the secondary metabolites of this fungus was identified as cladodionen, a known natural product from *Cladosporium* and an inseparable hybrid polyketide of two E/Z geometric isomers [17,18,19]. The inhibitory effect of cladodionen on QS has not been reported; as such, the in vitro activity against QS systems of *P. aeruginosa* was evaluated in this study.

## 2. Results

### 2.1. Screening of Fungi and Identification of Active Compound

**Figure 1:**
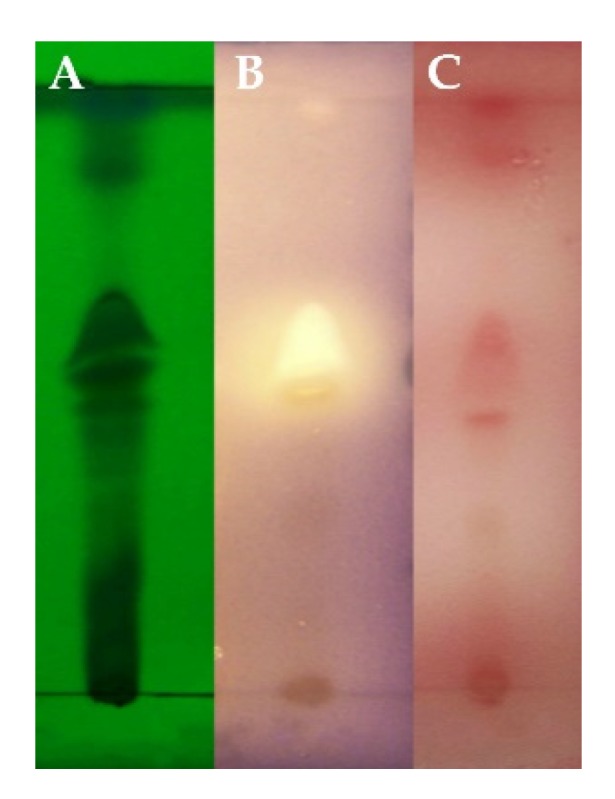
TLC-bioautography of secondary metabolites from *Cladosporium* sp. Z148. (**A**)TLC-UV determination; (**B**) TLC-bioautography with *C. violaceum* CV026 biosensor; (**C**) TLC-bioautography with *P. aeruginosa* QSIS-*lasI* biosensor.

A total of 235 strains of fungi were isolated from marine sediment collected in Jiaozhou Bay (China) (data not shown). The secondary metabolites of a marine fungus Z148 had inhibitory effects on QS (Figure 1). TLC-bioautography exhibited a white site with *Chromobacterium violaceum* CV026 biosensor and a red site with *P. aeruginosa* QSIS-*lasI* biosensor. The two active sites were in the same positions, suggesting that it could be the same compound that inhibited the QS systems of two biosensors. Based on 18S rDNA sequence analysis, the strain was identified and named *Cladosporium* sp. Z148. 

The fungus was cultured on a large scale, and 12.58 g of crude extract was isolated from 20 L of fermentation medium. Subfractions (S.20–S.35) obtained from Sephadex LH-20 chromatography had anti-QS activities; these subfractions were analyzed by high-performance liquid chromatography (HPLC) analysis. The results showed that it afforded a baseline separation of two chromatographic peaks (60% MeOH/H_2_O), but HPLC techniques failed to separate these two components. Based on the electrospray ionization mass spectrometry (ESIMS *m*/*z* 234.1 [M + H]^+^) and nuclear magnetic resonance (NMR) spectroscopic analyses (Figures S1, S2 and Table S1), the active compounds were identified as a pair of known isomers, cladodionen [17,18]. They were inseparable hybrid polyketide of two E/Z geometric isomers due to spontaneous and rapid isomerization between two forms (Figure S3). The purity of cladodionen from three subfractions (S.28–S.30) was more than 98% by HPLC analysis (70% MeOH/H_2_O) (Figure S4). These subfractions were used to evaluate anti-QS activity against *P. aeruginosa* PAO1 in vitro.

### 2.2. Growth Curve Analysis

**Figure 2:**
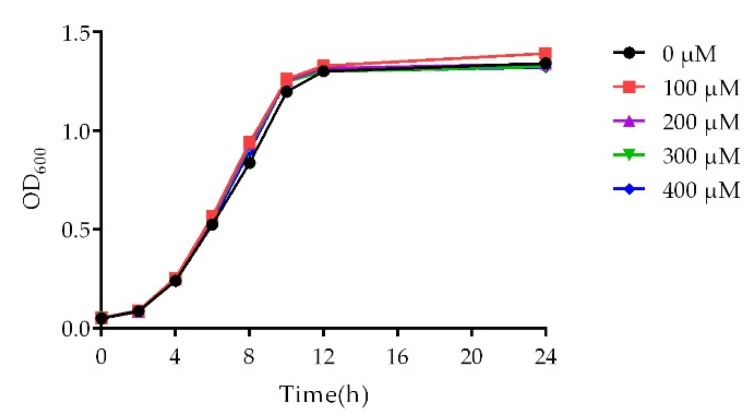
Effect of cladodionen on the growth curve of *P. aeruginosa* PAO1.

In order to eliminate the possibility that the decrease of bacterial population leads to the reduction of virulence factors production, the assay of virulence phenotypes should be carried out at subinhibitory concentrations. Therefore, a growth curve analysis was performed (Figure 2). There was no difference in the growth dynamics of *P. aeruginosa* PAO1 between the control group and cladodionen-treated groups ranging from 100 μM to 400 μM.

### 2.3. Effects of Cladodionen on the Production of Virulence Factors 

**Figure 3:**
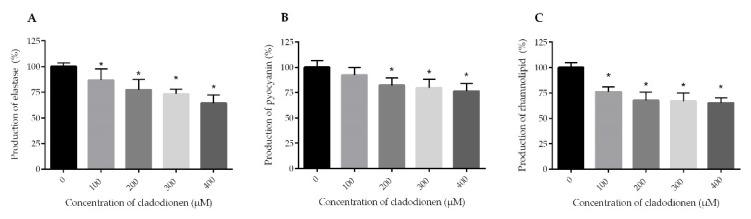
Effect of cladodionen on the production of virulence factors in *P. aeruginosa* PAO1. (**A**) Elastase; (**B**) Pyocyanin; (**C**) Rhamnolipid. *, *p* < 0.05.

*P. aeruginosa* uses virulence factors to infect and cause disease in patients. Elastase is the most abundant protein in *P. aeruginosa* secretomes; it modulates ion transport, immune response and tissue repair. The genes coding for the synthesis of elastase are regulated by the *las* system [6,20]. The production of elastase decreased in a dose dependent manner when the bacterial cultures were incubated with cladodionen at 100–400 μM (Figure 3A). Cladodionen inhibited the production of elastase by 35.7% at 400 μM. Pyocyanin, an extracellular redox active virulence factor of *P. aeruginosa*, exerts a pro-inflammatory effect and impairs ciliary function in patients with bronchiectasis and cystic fibrosis. Pyocyanin plays an important role in acute invasive infection and is mainly regulated by the *PQS* system [7]. The production of pyocyanin was inhibited by cladodionen, decreasing by 23.6% at 400 μM (Figure 3B). Rhamnolipid is a virulence factor which can cause rapid necrotic killing of polymorphonuclear leukocytes. It is mainly regulated by the *rhl* system of *P. aeruginosa* [6,21]. The yield of rhamnolipid in any of cladodionen-treated groups was lower than that of control group (Figure 3C). The production of rhamnolipid was inhibited by 34.7% when *P. aeruginosa* PAO1 was treated with 400 μM cladodionen. Cladodionen exhibited an inhibitory effect on virulence factors. Its effect on elastase and rhamnolipid was more significant than on pyocyanin. These results indicated that the *las* and *rhl* systems were more susceptible to cladodionen than the *PQS* system. 

### 2.4. Biofilm Assay 

**Figure 4:**
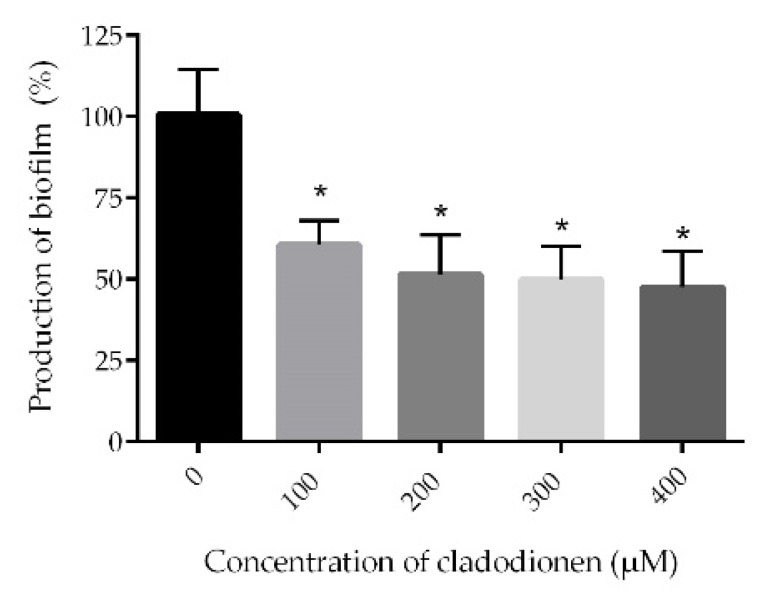
Effect of cladodionen on biofilm formation of *P. aeruginosa* PAO1. *, *p* < 0.05.

The severity of most of the *P. aeruginosa* infections in patients is correlated with biofilm formation. Moreover, biofilm formation is one of the mechanisms used by *P. aeruginosa* to develop drug resistant [2] related to QS systems [8]. The productions of biofilms decreased with increasing concentrations of cladodionen (Figure 4). Compared with the control group, cladodionen inhibited the production of biofilms by 52.4% at 400 μM. 

### 2.5. Swarming and Swimming Motility 

**Figure 5:**
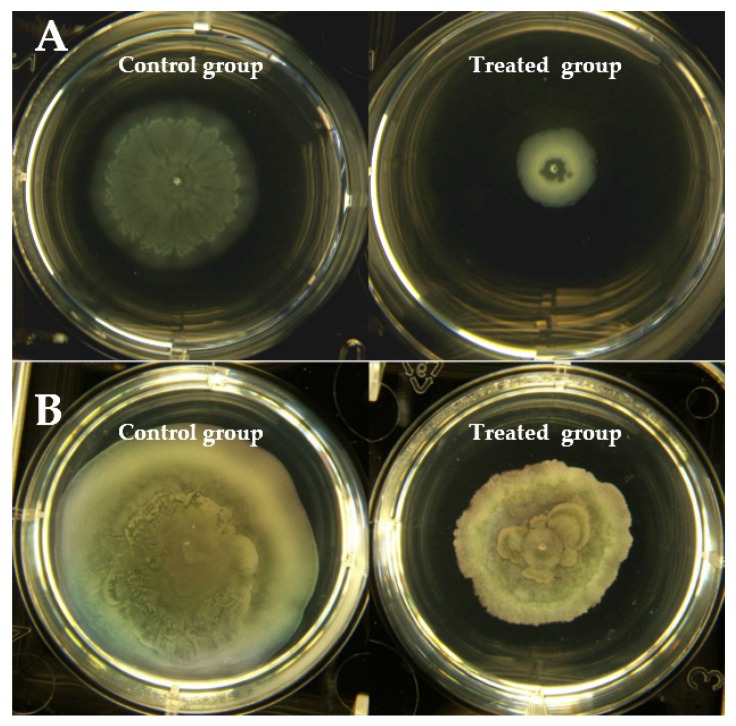
Effect of cladodionen on motilities of *P. aeruginosa* PAO1. (**A**) Swarming motility; (**B**) Swimming motility.

Motility has been shown to be associated with the virulence of *P. aeruginosa*. It plays an important role in mobilization to and colonization of a variety of environments, adhesion to substrates and biofilm formation in *P. aeruginosa* [22]. The swarming motility is closely related to the *rhl* system [23], and the swimming motility is associated with the *las* and *rhl* systems [24]. Cladodionen was capable of inhibiting motilities of *P. aeruginosa* (Figure 5). The results of the swarming motility showed that the branches of the control group were longer and denser than those of the treated group (Figure 5A). In the control group, a growing colony swam towards the edge of the dish, covering the entire distance, while impaired swimming motility was observed in the treated group (Figure 5B).

### 2.6. Real-Time RT-PCR

**Figure 6:**
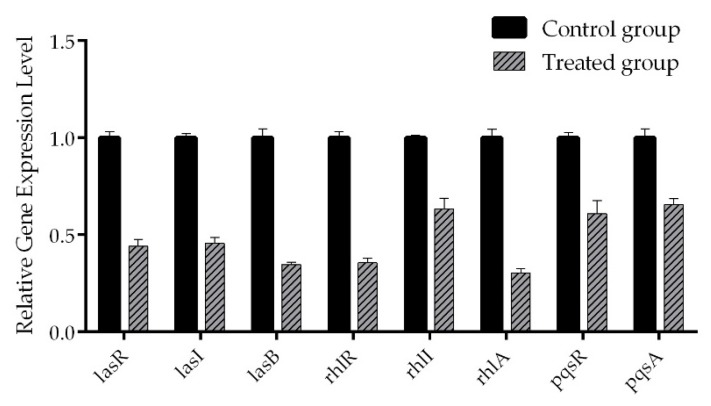
The effect of cladodionen on the mRNA expressions of QS-related genes in *P. aeruginosa* PAO1.

In consideration of the ability of cladodionen to inhibit virulence factors, biofilm formation and motilities, the mRNA expressions of several QS-related genes in *P. aeruginosa* were detected by real-time RT-PCR because of their key role in virulence expression. These genes are involved in receptor proteins (*lasR*, *rhlR* and *pqsR*), autoinducer synthases (*lasI*, *rhlI* and *pqsA*) and virulence factors including elastase and rhamnolipid (*lasB* and *rhlA*). The mRNA expressions of QS-related genes were down-regulated by cladodionen at 400 μM (Figure 6). The mRNA expressions of *lasR, lasI and lasB* were down-regulated by 55.9%, 54.3% and 65.3%, respectively; the mRNA expressions of *rhlR, rhlI and rhlA* were down-regulated by 64.4%, 36.6% and 69.6%, respectively; the mRNA expressions of *pqsR* and *pqsA* were down-regulated by 39.0% and 34.4%, respectively. The results showed that cladodionen had inhibitory activity on the QS systems of *P. aeruginosa*, especially the *las* and *rhl* systems. 

### 2.7. Molecular Docking Analysis

**Figure 7:**
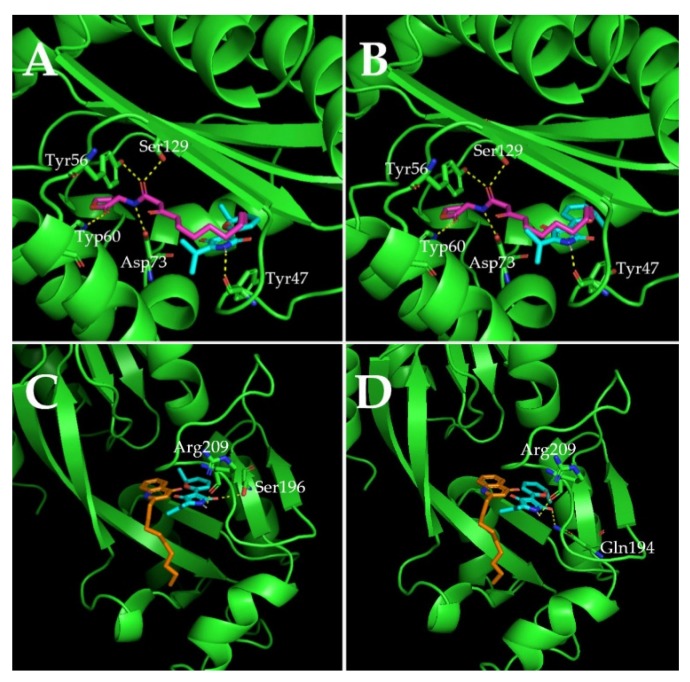
Docked complex of the QS receptor proteins with their natural ligands and cladodionen. (**A**) LasR bound to 3-oxo-C12-HSL and cladodionen(a); (**B**) LasR bound to 3-oxo-C12-HSL and cladodionen(b); (**C**) PqsR bound to NHQ and cladodionen(a); (**D**) PqsR bound to NHQ and cladodionen(b). QS receptor proteins are indicated with green. Blue, purple and orange sticks represent cladodionen, 3-oxo-C12-HSL and NHQ, respectively. The hydrogen bonds are shown as yellow dotted lines.

**Figure 8:**
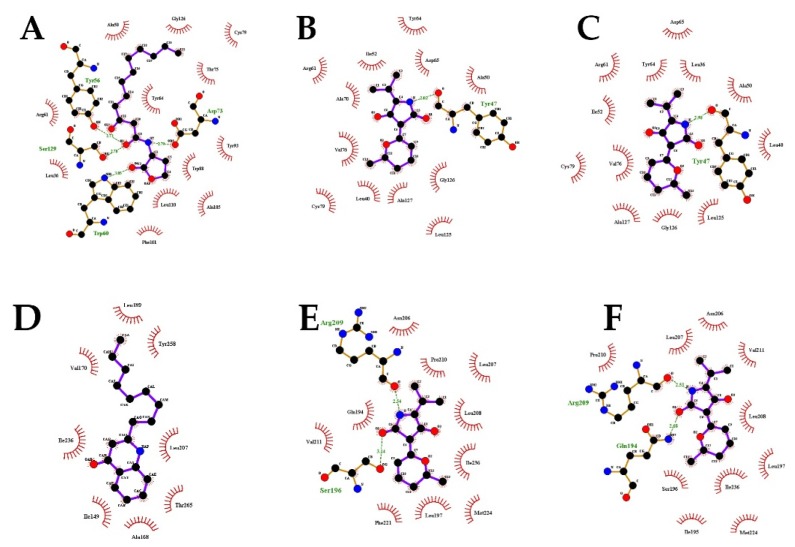
Interactions between QS receptor proteins and various ligands. (**A**) LasR bound to 3-oxo-C12-HSL; (**B**) LasR bound to cladodionen(a); (**C**) LasR bound to cladodionen(b); (**D**) PqsR bound to NHQ; (**E**) PqsR bound to cladodionen(a); (**F**) PqsR bound to cladodionen(b).

In order to study the inhibitory mechanism of cladodionen on QS systems, the possibility of binding interactions between cladodionen and QS receptor proteins was explored by molecular docking analysis. The study was performed using two QS receptor proteins, i.e., LasR and PqsR, bound with AHLs and quinolone signal molecules, respectively. The interactions of the natural ligand 3-oxo-C12-HSL in LasR and of natural ligand NHQ in PqsR were regarded as controls for molecular docking analysis, and were highly consistent with those reported in X-ray structures [11,12,13]. Cladodionen bound to the QS receptor proteins by hydrogen bonding interactions and hydrophobic interactions (Figure 7, Figure 8, Table 1 and Table 2). The docking energies of QS receptor proteins and cladodionen were lower than those of QS receptor proteins and their natural ligands, indicating that cladodionen could form stable complexes with QS receptors, and that it had better binding affinity to LasR and PqsR than natural ligands. The docking energy of cladodionen and LasR was lower than that of cladodionen and PqsR, indicating that the binding affinity of cladodionen to LasR was higher than to PqsR. There is no crystal structure for RhlR. Based on similarity, the interactions of LasR with its natural ligand and cladodionen are expected to extend to RhlR. Cladodionen is a pair of isomers, and the only significant differences between the two configurations (a and b) were the chemical shifts of C-2 and C-4 (Table S1). The two configurations of cladodionen were analyzed and compared with each other. The docking energies of cladodionen(a) and QS receptor proteins were slightly lower than those of cladodionen(b) and QS receptor proteins. Cladodionen(a) and cladodionen(b) interacted with the same QS receptor sites, except for amino acid residues of LasR (Ala70/Leu36) in the key hydrophobic interactions and PqsR (Ser196/Gln194 and Phe221/Ile195) in the hydrogen bonding or key hydrophobic interactions. 

## 3. Discussion

In order to treat *P. aeruginosa* infections, interfering with QS systems is an emerging strategy to abolish *P. aeruginosa* pathogenicity without affecting bacterial growth. Cladodionen, an inhibitor of QS systems in *P. aeruginosa*, was isolated from a marine fungus *Cladosporium* sp. Z148. The inhibitory effect of cladodionen on the production of virulence factors, the formation of a biofilm and motilities of *P. aeruginosa* PAO1 were determined at subinhibitory concentrations. The mechanism by which cladodionen inhibits QS systems was preliminarily explored by detecting mRNA expressions of QS-related genes and molecular docking analysis. 

Cladodionen was first isolated from marine fungus *Cladosporium* sp. OUCMDZ-1635, and had cytotoxic activities against a variety of human cancer cell lines [17]. Cladodionen belongs to cladosporiumins compounds which are composed of 2,4-pyrrolidinedione (tetramic acid) and a valine residue [17,19]. The synthetic pathway of cladosporiumins includes polyketide synthase (PKS) and nonribosomal peptide synthase (NRPS) [19]. The anti-QS activity of cladosporiumins has not been reported before. In addition, only five compounds, i.e., apiodionen, bripiodionen, vermelhotin, hypoxyvermelhotins and cladosporiumin I(1) [17,18], have the similar skeletons to cladodionen, comprising 2,4-pyrrolidinedione and a pyrene ring or a dihydropyran ring by a double bond. The anti-QS activity of the aforementioned compounds has not yet been investigated. This is the first report on the anti-QS effect of cladodionen.

Cladodionen had a better inhibitory effect than pyocyanin on the production of elastase and rhamnolipid. Elastase, rhamnolipid and pyocyanin are mainly regulated by the *las*, *rhl* and *PQS* systems, respectively [6,7]. Therefore, cladodionen had a stronger inhibitory effect on the *las* and *rhl* systems than on the *PQS* system. The results of real-time RT-PCR also confirmed that the *las* and *rhl* systems were more susceptible to cladodionen than the *PQS* system. 

The QS system is a key regulator of biofilm formation. The biofilm of the *lasI* mutant is much thinner than that of wild-type organism [8]. Rhamnolipid could be used as a surfactant to maintain the structure of biofilm [9]. PqsE and RhlR comprise an autoinducer synthase–receptor pair which could influence biofilm development [10]. The regulation of QS systems on biofilms is complex. The reduction of virulence factors and the down-regulation of the mRNA expression of genes related to the *las* and *rhl* systems suggested that cladodionen mainly inhibited biofilm formation by interfering with the *las* and *rhl* systems. 

*P. aeruginosa* is unable to swarm in the *rhl* mutant, which could not produce rhamnolipid surfactant [23], and the *las* and *rhl* systems play an important role in swimming motility [24]. Cladodionen could inhibit swarming and swimming motility by repressing the *las* and *rhl* systems. The presence of cladodionen contributed to a decrease of rhamnolipid surfactant; this was an important reason for the inhibition of swarming motility. Besides QS systems, flagella and pili are necessary for motilities [23]. The effect of cladodionen on flagella and pili is uncertain and needs to be further studied.

Cladodionen bound more easily to LasR and PqsR than natural ligands, suggesting that cladodionen inhibited the QS systems in *P. aeruginosa* by competing with the natural ligands. Although hydrogen bonding interactions were different, Ala50, Cys79, Tyr64, Leu36 and Arg61 were the key amino acid residues taking part in similar hydrophobic interactions with LasR between cladodionen and 3-oxo-C12-HSL. The interactions between NHQ and PqsR were all hydrophobic, while the hydrogen bonding and hydrophobic interactions were both important for the interactions of cladodionen and PqsR. Additionally, only two residues of PqsR (Ile236 and Leu207) interacted with cladodionen and NHQ at the same time. The above difference of interaction modes and binding sites may be an important reason why the docking energy of cladodionen and LasR was lower than that of cladodionen and PqsR. The interactions between natural ligands and QS receptors were highly consistent with those reported in X-ray structures [11,12,13], indicating that the molecular docking-derived models possessed a certain degree of reliability. However, the semi flexible docking method used in this work might cause some interactions to go undetected due to the limitation of inflexible proteins. This might be one of the reasons why cladodionen interacted with the receptor proteins quite differently compared to the natural ligands. Therefore, further research is needed to elucidate the interactions between ligands and flexible proteins.

The tetramic acid scaffold is an important structure which has been found in terrestrial and marine organisms [25]. In addition to cladodionen, similar structures have been found in QSIs, such as tenuazonic acid [26] and equisetin [27]. Tenuazonic acid inhibits QS of *C. violaceum* CV017 without interfering with bacterial vitality [26]. Equisetin inhibits three QS systems of *P. aeruginosa*; it has the strongest inhibitory effect on the *PQS* system [27]. The anti-QS activity of the other compounds with similar skeletons to cladodionen, and other cladosporiumins, should be explored. It is important to study the structure–activity relationships among these chemicals to illustrate the modes of action, optimize the structures and improve anti-QS activity. 

## 4. Materials and Methods

### 4.1. Strains and Culture Conditions

*C. violaceum* CV026, *P. aeruginosa* QSIS-*lasI* and *P. aeruginosa* PAO1 were cultured for 12 h at 160 rpm in 5 mL LB medium: 1.0% *w*/*v* NaCl, 1% *w/v* tryptophan and 0.5% *w/v* yeast extract. *C. violaceum* CV026 was cultured at 30 °C and the other strains were cultured at 37 °C. The overnight cultures of *P. aeruginosa* PAO1 were diluted with fresh LB medium to OD_600_ ≈ 0.05, cultivated for 2 h and then incubated with cladodionen at 0–400 μM for another 6 h to detect the production of virulence factors. OD_600_ was measured and 1 mL of bacterial culture was centrifugated at 12000 rpm for 2 min to get the supernatant.

### 4.2. Isolation of Marine Fungi and Preparation of the Crude Extracts

The fungal strains were obtained from marine sediment collected in Jiaozhou Bay, China. The fungi were isolated and grown on fungal medium (2.0% *w/v* mannitol, 2.0% *w/v* maltose, 1.0% *w/v* glucose, 0.05% *w/v* KH_2_PO_4_, 0.03% *w/v* MgSO_4_, 0.1% *w/v* corn flour, 0.3% *w/v* yeast extract, 3.33% *w/v* sea salt and 2% *w/v* agar) at 30 °C for 7 days. The fermentation products extracted with ethyl acetate (EtOAc) were filtered using 0.22 μM membranes, freeze-dried and redissolved with methanol to 50 mg/mL. 

### 4.3. Screening for QSIs

The crude extracts were screened for anti-QS properties by TLC-bioautography with *C. violaceum* CV026 biosensor or *P. aeruginosa* QSIS-*lasI* biosensor as described previously [28]. One microliter of crude extracts (50 mg/mL) was applied by TLC performed on silica gel plates and developed using CH_2_Cl_2_/MeOH (15:1, *v/v*). The plates were checked under UV light and overlaid with *C. violaceum* CV026 assay agar culture (15 mL LB agar medium, bacterial culture (OD_600_ ≈ 0.015), 80 μg/mL kanamycin and 530 nM C6-HSL) or *P. aeruginosa* QSIS-*lasI* assay agar culture (20 mL LB agar medium, bacterial culture (OD_600_ ≈ 0.015), 0.75 mg/mL 2,3,5-triphenyltetrazolium chloride, 80 μg/mL gentamicin and 60 nM 3-oxo-C12-HSL). The TLC overlays were incubated overnight at 30 °C (*C. violaceum* CV026) or 37 °C (*P. aeruginosa* QSIS-*lasI*).

### 4.4. Species Identification of Selected Fungus

Among the 235 fungi screened for anti-QS potential, fungus Z148 was selected for species identification based on its anti-QS activity against *C. violaceum* CV026 and *P. aeruginosa* PAO1. The selected fungus was identified by 18S region sequencing using primers NS1 (5′-GTAGTCATATGCTTGTCTC-3′) and NS8 (5′-TCCGCAGGTTCACCTACGGA-3′). The amplified products were sequenced in Beijing Ruibo Xingke Biotechnology Co. Ltd (Beijing, China). From 18S sequencing and comparison of the obtained sequence from NCBI, the selected fungus was identified and named *Cladosporium* sp. Z148. The sequence was submitted to the NCBI GenBank database (accession number MT093347).

### 4.5. Purification of QSIs

Fermentation of *Cladosporium* sp. Z148 was conducted in 20 L of fungal medium as described at 30 °C for 15 days. Solid fermentation products were extracted with an equal volume of EtOAc and the combined EtOAc extracts were dried in vacuo to yield 12.58 g of crude extract. TLC-bioautography was adopted as a bioassay-guided isolation approach to identify the active fractions. The crude extract was fractionated by a silica gel gradient vacuum liquid chromatography column, eluting with a step gradient of CH_2_Cl_2_/MeOH from 70:1 to 35:1 (*v/v*), and 28 fractions (Fr.1–Fr.28) were collected. Fractions 2 and 3 were subjected to Sephadex LH-20 chromatography with MeOH to afford 114 subfractions (S.1–S.114). Subfractions were analyzed by TLC (CH_2_Cl_2_/MeOH, 15:1, *v/v*) and 16 subfractions (S.20–S.35) had anti-QS activity. The purity of active compounds from subfractions was determined by HPLC analysis on a C18 reverse phase chromatographic column.

Cladodionen: Yellow powder, ^1^H and ^13^C NMR data, see Table S1; ESIMS *m*/*z* 234.1 [M + H]^+^.

### 4.6. Growth Curve Assay

The overnight bacterial culture of *P. aeruginosa* PAO1 was diluted to an OD_600_ of 0.05 with fresh LB medium and transferred into a 96-well plate (200 μL/well) exposed to cladodionen at 0–400 μM. The plate was incubated for 24 h at 90 rpm and 37 °C. The growth state of the bacteria was observed and the growth curve of PAO1 was drawn from the measured OD_600_. 

### 4.7. Effects of Cladodionen on the Production of Virulence Factors

#### 4.7.1. Elastase Activity Assay

A modified method, as previously reported, was used to determine elastase activity [12]. The supernatant (400 μL) was collected by a 0.22 μm nylon filter, and then 3 mg Congo red and 800 μL buffer (100 mM Tris-HCI/1 mM CaCl_2_, pH = 7.2,) were added. After incubation at 160 rpm and 37 °C for 6 h, the samples were centrifuged at 12000 rpm for 2 min and 200 μL of the supernatant was measured at a wavelength of 490 nm. 

#### 4.7.2. Pyocyanin Assay

The method of pyocyanin assay was described previously [12]. The specific experimental process was as follows: The supernatant (800 μL) was extracted with chloroform (600 μL), the organic layer (500 μL) was incubated with 0.2 M HCl (200 μL) for 30 min at 37 °C, and 200 μL of the water layer was measured at a wavelength of 520 nm. 

#### 4.7.3. Rhamnolipid Assay

The previously reported method [29] for the determination of rhamnolipid was slightly modified and applied here. The supernatant (800 μL) was extracted with diethyl ether (1 mL) and 800 μL of the organic layer was allowed to evaporate. Residues were dissolved in a solution of 100 μL sterile water, 700 μL 70% H_2_SO_4_ and 100 μL 1.6% orcinol. The solution was incubated at 80 °C for 30 min and measured at a wavelength of 420 nm.

### 4.8. Biofilm Assay

A biofilm detection method described previously [30] with minor modifications was used as follows: The overnight bacterial culture was diluted 1:10000 in fresh LB medium, and 200 μL of each dilution was added to a 1.5-mL Eppendorf tube with cladodionen at 0–400 μM. The samples were allowed to grow for 12 h (at 37 °C under static condition), and growth was measured at 600 nm. The tubes were rinsed three times with phosphate-buffered saline (PBS) to eliminate nonadherent cells. The biofilm was then stained with 0.1% crystal violet (200 μL) for 15 min, after which the stained biofilm was rinsed with PBS three times to remove any unbound dye. The crystal violet bound to the biofilm was dissolved with 400 μL 33% (*v/v*) acetic acid and the absorbance was measured at a wavelength of 590 nm.

### 4.9. Swimming and Swarming Motility Assay

The effect of cladodionen on the *P. aeruginosa* PAO1 swarming motility was tested as described previously [31]. The swarming agar medium consisted of M9 salt medium [23] supplemented with 1 mM MgSO_4_, 1 mM CaCl_2_, 1.1 mM glucose, 0.5% *w/v* casamino acids, 1.8 μM FeSO_4_ and 0.5% *w/v* agarose. The swimming agar medium consisted of 1% *w/v* tryptone, 0.5% *w/v* NaCl and 0.3% *w/v* agarose [32]. Cladodionen was added into the above mediua at a final concentration of 400 μM. The overnight culture (1 mL) was centrifugated at 4000 rpm for 5 min and concentrated to 200 μL. Plate inoculation was carried out by spotting a 1 μL drop of concentrated culture at the center of the swarming and swimming plates and incubating for 12 h. 

### 4.10. Real-Time RT-PCR

The effect of cladodionen on the mRNA expression of QS-related genes in *P. aeruginosa* PAO1 was detected as described previously [27]. RNA was isolated with a bacteria RNA isolation kit (Nobelab Biotechnologies, Beijing, China). Reverse transcription was performed from 1 μg of RNA using HiScript III RT SuperMix (Vazyme Biotech, Nanjing, China). Amplification was performed with ChamQ Universal SYBR qPCR Master Mix (Vazyme Biotech, Nanjing, China). The cycling conditions were 95 °C for 10 min followed by 40 cycles of denaturation at 95 °C for 15 s, annealing and extension at 60 °C for 60 s. The *rpsl* gene was used as the internal reference for normalizing gene expression. All primer sequences for real-time RT-PCR are listed in Table S2. 

### 4.11. Molecular Docking Analysis

A molecular docking analysis was performed as described in a previous report [33]. The ChemBioOffice 2018, Autodock 1.5.6, PyMol 2.3.0 and LigPlot^+^ v.1.4 software programs were used for molecular docking analysis. The receptor proteins LasR (PDB ID, 3IX3) and PqsR (PDB ID, 4JVD) were downloaded from the protein data bank (PDB). Cladodionen was drawn and its energy was minimized with ChemBio3D Ultra 18.0. The natural ligands were obtained from QS receptors (3IX3 and 4JVD) by PyMol 2.3.0. All water molecules and cocrystalized ligands of QS receptors (3IX3 and 4JVD) were removed. The proteins were further modified by incorporating new polar hydrogens, and the Gasteiger charges were computed. The ligands were assigned with hydrogens and Gasteiger charges, followed by definition of the rotatable bonds. Docking grid sites were set up around their natural autoinducer binding sites. The Lamarckian genetic algorithm was used as a parameter for molecular docking, and the results of the docking computations were ranked by binding energy. The PyMol molecular graphic system and LigPlot^+^ were used to visualize conformations and interactions between ligands and receptor proteins.

### 4.12. Statistical Analysis

Data were analyzed on SPSS 13.0 software using ANOVA with Bonferonni tests. A *p*-value of less than 0.05 was considered significant for differences between control group and treated groups. GraphPad Prism 6 was used for graph construction. Real-time RT-PCR was performed in duplicate and the other assays were performed in triplicate. 

## 5. Conclusions

Cladodionen, an inhibitor of QS systems in *P. aeruginosa*, was isolated from marine fungus *Cladosporium* sp. Z148, obtained from Jiaozhou Bay (China). Cladodionen had a better inhibitory effect than pyocyanin on the production of elastase and rhamnolipid. The formation of biofilms and motilities were also inhibited by cladodionen. Based on the results of real-time RT-PCR, the *las* and *rhl* systems were more susceptible to cladodionen than the *PQS* system. Molecular docking analysis showed that cladodionen had better binding affinity to LasR and PqsR than natural ligands. Compared with the *PQS* system, cladodionen had a more significant inhibitory effect on the *las* and *rhl* systems. This is the first report on the anti-QS effect of cladodionen. The structure–activity relationships among cladodionen and its structurally related compounds should be further studied to illustrate the modes of action, optimize the structures and improve anti-QS activity. 

## Figures and Tables

**Table 1:** Details of the docked complex of the LasR with 3-oxo-C12-HSL and cladodionen.

Molecule	Docking Energy (kcal/mol)	Hydrogen Bonding Interactions	Key Hydrophobic Interactions
3-oxo-C12-HSL	−5.47	Ser129, Tyr56, Asp73, Trp60	Ala50, Gly126, Cys79, Thr75, Tyr64, Tyr93, Trp88, Leu110, Ala105, Phe101, Leu36, Arg61
Cladodionen(a)	−7.38	Tyr47	Ala70, Val76, Cys79, Leu40, Ala127, Leu125, Gly126, Ala50, Asp65, Tyr64, Ile52, Arg61
Cladodionen(b)	−7.04	Tyr47	Leu36, Val76, Cys79, Leu40, Ala127, Leu125, Gly126, Ala50, Asp65, Tyr64, Ile52, Arg61

**Table 2:** Details of the docked complex of the PqsR with NHQ and cladodionen.

Molecule	Docking Energy (kcal/mol)	Hydrogen Bonding Interactions	Key Hydrophobic Interactions
NHQ	−5.22	/	Val170, Ile236, Ile149, Ala168, Thr265, Leu207, Tyr258, Leu189
Cladodionen(a)	−6.85	Arg209, Ser196	Gln194, Phe221, Val211, Leu197, Met224, Ile236, Leu208, Leu207, Pro210, Asn206
Cladodionen(b)	−6.81	Arg209, Gln194	Ser196, Ile195, Val211, Leu197, Met224, Ile236, Leu208, Leu207, Pro210, Asn206

## Supplementary Materials

The following are available online at https://www.mdpi.com/1660-3397/18/4/205/s1, Figure S1: ^1^H-NMR spectroscopy of cladodionen (Solvent: DMSO-d6), Figure S2: ^13^C-NMR spectroscopy of cladodionen (Solvent: DMSO-d6), Figure S3: Chemical structure of cladodionen, Figure S4: HPLC analysis of cladodionen, Table S1: ^1^H and ^13^C NMR data for cladodionen, Table S2: The primer sequences for real-time RT-PCR.
