# Supplementary material for: Cladodionen Is a Potential Quorum Sensing Inhibitor Against Pseudomonas aeruginosa
Source: Mar Drugs. 2020 Apr 10;18(4):205. doi: 10.3390/md18040205 (PMC7230538; doi:10.3390/md18040205)
Supplement: Supplementary file 1 [file marinedrugs-18-00205-s001.pdf]

Supporting Information

Cladodionen, a Potential Quorum Sensing Inhibitor Against *Pseudomonas aeruginosa*

Mengjia Wang [1,2,3], Lu Zhao [1,2,3], Hao Wu [1,2,3], Chaoyue Zhao [1,2,3], Qianhong Gong [1,2,3,*] and Wengong Yu [1,2,3,*]

[1] School of Medicine and Pharmacy, Ocean University of China, 5 Yushan Road, Qingdao 266003, China; wangmengjia6877@stu.ouc.edu.cn (M.W.); zhaolu@stu.ouc.edu.cn (L.Z.); wuhao1330@stu.ouc.edu.cn (H.W.); zhaochaoyue2712@stu.ouc.edu.cn (C.Z.)

[2] Laboratory for Marine Drugs and Bioproducts, Qingdao National Laboratory for Marine Science and Technology, 1 Wenhai Road, Qingdao 266237, China

[3] Provincial Key Laboratory of Glycoscience and Glycotechnology, Ocean University of China, 5 Yushan Road, Qingdao 266003, China

* Correspondence: gongqh@ouc.edu.cn (Q.G.); yuwg66@ouc.edu.cn (W.Y.); Tel: +86-532-82032067 (Q.G.); +86-532-82031680 (W.Y.)

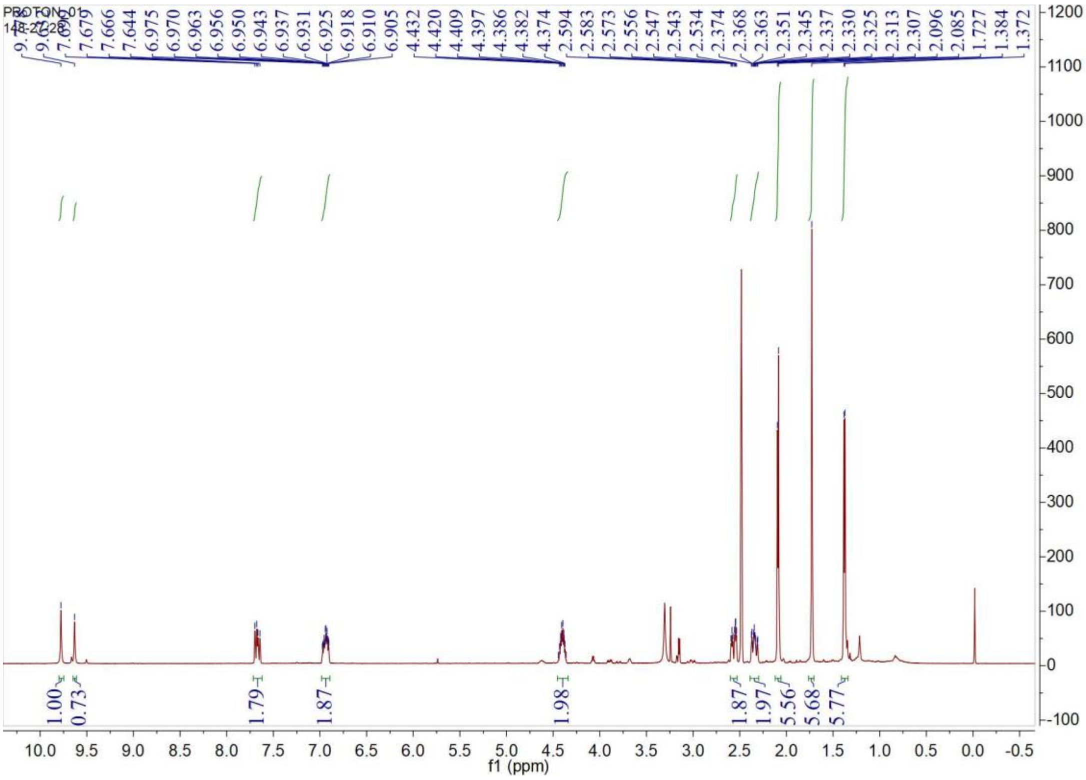

Figure S1. ^{1}H-NMR spectroscopy of the anti-QS compound (Solvent: DMSO-d6).

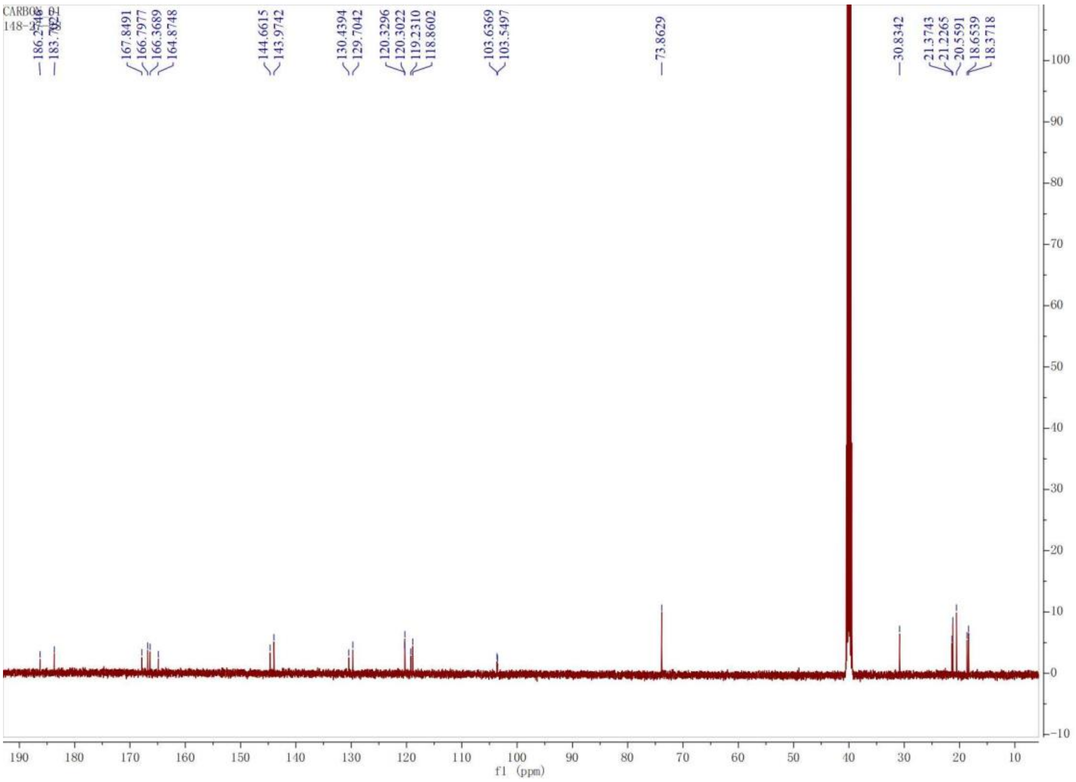

Figure S2. ^{13}C-NMR spectroscopy of the anti-QS compound (Solvent: DMSO-d6).

Table S1. ^{1}H and ^{13}C NMR Data for cladodionen.

No.	a		b	
	δC	δH, Mult. (*J* in Hz)	δC	δH, Mult. (*J* in Hz)
1	-	9.77, s	-	9.63
2	166.80		164.9	
3	103.6		103.5	
4	183.7		186.3	
5	129.7		130.4	
6	166.3		167.8	
7	120.3	7.70, d	120.3	7.67, d
8	144.0	6.96, m	144.7	6.97, m
9	30.8	2.35, m; 2.55, m	30.8	2.33, m; 2.58, m
10	73.9	4.40, m	73.9	4.39, m
11	20.6	1.38, d	20.6	1.37, d
12	118.9		119.2	
13	21.2	1.73, s	21.3	1.73, s
14	18.7	2.09, s	18.4	2.10, s

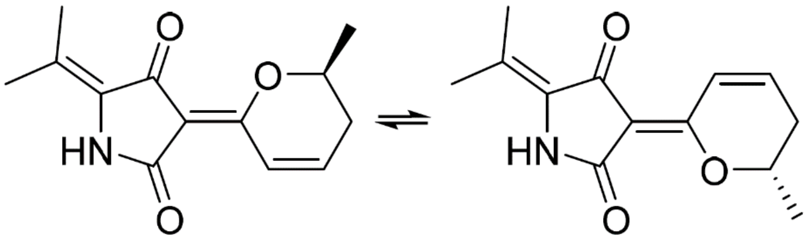

Figure S3. Chemical structure of cladodionen.

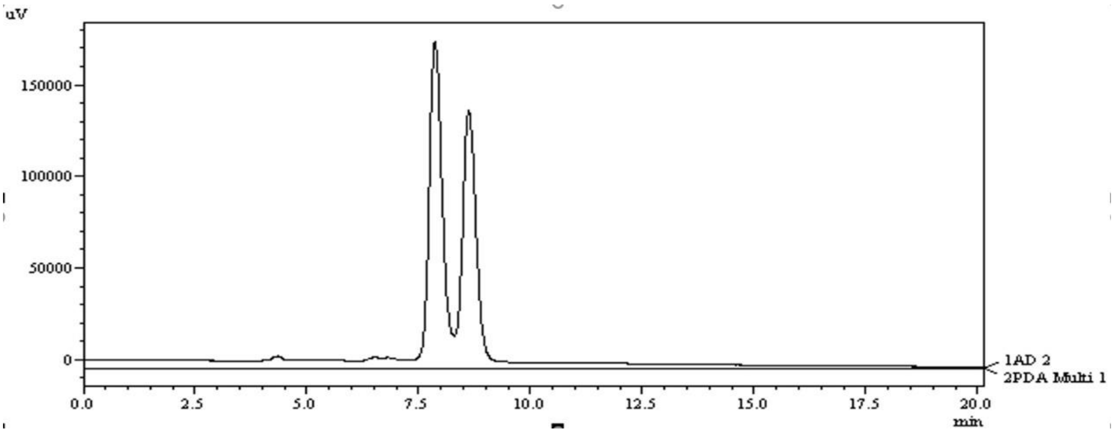

Figure S4. HPLC analysis of cladodionen.

Table S2. The primer sequences for real-time RT-PCR.

Primers	sequences (5′ to 3′)	primers	sequences (5′ to 3′)
Rpsls (F)	GCAACTATCAACCAGCTG GTG	*Rpsls* (R)	GCTGTGCTCTTGCAGGTTGT G
lasR (F)	ACGCTCAAGTGGAAAATT GG	*lasR* (R)	TCGTAGTCCTGGCTGTCCTT
lasI (F)	GGCTGGGACGTTAGTGTC AT	*lasI* (R)	AAAACCTGGGCTTCAGGAG T
lasB(F)	ACCAGAAGATCGGCAAG TAC	*lasB*(R)	GTTGACCTGCTTGTAGGTGT TG
rhlR (F)	CTGGGCTTCGATTACTA CGC	*rhlR* (R)	CCCGTAGTTCTGCATCTGGT
rhlI (F)	AAGGACGTCTTCGCCTAC CT	*rhlI* (R)	GCAGGCTGGACCAGAATAT C
pqsR(F)	CTGATCTGCCGGTAATTG G	*pqsR*(R)	ATCGACGAGGAACTGAAGA
pqsA (F)	GACCGGCTGTATTCGATT C	*pqsA* (R)	GCTGAACCAGGGAAAGAA C
